# Short-wave near-infrared emissive GdPO_4_:Nd^3+^ theranostic probe for *in vivo* bioimaging beyond 1300 nm†

**DOI:** 10.1039/c7ra12864a

**Published:** 2018-04-04

**Authors:** Qiuhua Yang, Xiaolong Li, Zhenluan Xue, Youbin Li, Mingyang Jiang, Songjun Zeng

**Affiliations:** College of Physics and Information Science, Key Laboratory of Low-dimensional Quantum Structures and Quantum Control of the Ministry of Education, Synergetic Innovation Center for Quantum Effects and Applications, Hunan Normal University Changsha 410081 Hunan China songjunz@hunnu.edu.cn

## Abstract

The optical probes working in the second near-infrared (NIR-II) window have attracted increasing research interest for their advantages of high tissue penetration depth, low autofluorescence, and unprecedentedly improved imaging sensitivity and spatial resolution. Therefore, it is of great significance to design a new nanoplatform by integration of NIR-II optical imaging and drug delivery functions. Herein, a multifunctional nanoplatform based on GdPO_4_:Nd^3+^ yolk–shell sphere was developed for dual-modal *in vivo* NIR-II/X-ray bioimaging and pH-responsive drug delivery. The *in vivo* NIR-II bioimaging and real-time tracking presented that these probes were mainly accumulated in liver and spleen. Moreover, owing to the large X-ray absorption coefficient of Gd^3+^, these probes are successfully used as superior X-ray imaging agents than iobitridol. The *in vivo* toxicity assessments demonstrate the low biotoxicity of the GdPO_4_:Nd^3+^ spheres in living animals. More importantly, apart from the excellent dual-modal bioimaging, these yolk–shell-structured probes were also used as ideal nanotransducer for pH-responsive drug delivery of doxorubicin (DOX). These findings open up the opportunity of designing theranostic nanoplatform with integration of imaging-based diagnosis and therapy.

## Abstract 2

A multifunctional theranostic nanoplatform based on GdPO_4_:Nd^3+^ yolk–shell sphere was developed for dual-modal *in vivo* NIR-II/X-ray bioimaging and pH-responsive drug delivery.

## Graphical abstract

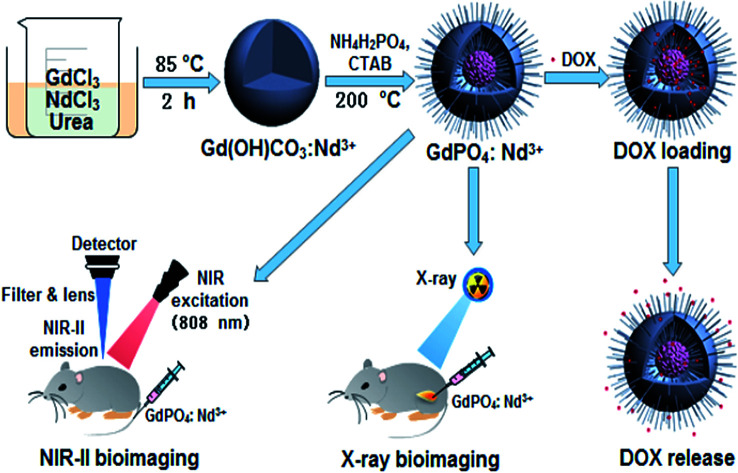

## 1. Introduction

Recently, the development of optical probes has opened up the new field of bioimaging and promoted its applications for the early detection and diagnosis of diseased tissues, due to its advantages of high accuracy, high sensitivity, fast feedback and absence of radiation.^1–5^ However, optical probes with short wavelength emissions such as the visible light region hold large absorption efficient and scattering losses in biotissues (blood, hemoglobin and lipids), limiting their imaging efficiency and tissue penetration depth.^6,7^ Typically, the optical probes that emitting in the visible range (400–750 nm) can only penetrate approximately 1 mm into the tissue.^8,9^ Fortunately, the penetration depth and imaging sensitivity can be improved by using the optical probes capable of emitting in the first near-infrared (NIR-I) window (750–900 nm) owing to the reduced autofluorescence and scattering losses.^10–14^ However, the imaging sensitivity, spatial resolution, and penetration depth of NIR-I emitting probes are still limited. Therefore, it is an urgent task to design an optical probe with remarkably reduced absorption and scattering losses in biotissues.

Significantly, in recent years, optical bioimaging achieved in a new second near-infrared (NIR-II, 1000–1700 nm) biological window has attracted enormous attention, due to its lower absorbance, tissue autofluorescence and up to a 1000-fold reduction in scattering losses than the NIR-I.^15,16^ So far, there are several kinds of materials capable of generating NIR-II light, such as NIR-II-emitting semiconducting quantum dots (QDs),^17,18^ single-walled carbon nanotubes (SWNTs)^19–22^ and molecular fluorophores.^23,24^ However, NIR-II-emitting QDs always comprise toxic elements, such as lead, mercury and arsenic, making it unacceptable for *in vivo* bioimaging.^25,26^ Although NIR-II-emitting Ag_2_S QDs revealed low toxicity, seriously quenching effect under both air and irradiation and low quantum yield (QY) remarkably limited their applications in bioimaging area.^8,27^ Despite SWNTs have achieved great progress in NIR-II bioimaging, their low QY, broad-band emission peak (greater than 300 nm) and a large length distribution make it difficult for multi-spectral imaging with non-overlapping signals, and clarification of its pharmacokinetic behaviour.^16,19,21,28^ Hence, it is meaningful to design a probe working in NIR-II window with a lower toxicity, narrower emission band, controlled size, and higher QY.

In comparison, rare earth-doped luminescent materials have great promise for deep tissue bioimaging in the NIR-II window, due to its high efficiency, low long-term cytotoxicity, low photo-bleaching, and long luminescence lifetimes.^16,29–32^ To date, Nd-based complexes or nanoparticles were primarily reported as NIR-II fluorescence bioimaging agents, owing to its efficient luminescence at 900, 1050, and 1330 nm under the excitation of the 808 nm laser.^12,29,31^ Moreover, the absorption of 808 nm light in water is much lower than that at 980 nm, subsequently minimizing the overheating effect usually induced by the conventional 980 nm excitation.^5,33^ Therefore, the development of rare earth-based optical probes working in the NIR-II window under the excitation of the 808 nm laser, can achieve unprecedented improvements in detection depth and resolution owing to their unparalleled advantages as stated above.

On the other hand, according to the previous reports,^34–41^ the hollow-typed materials have promising applications in drug delivery, owing to their unique properties of larger specific area, lower density, strong encapsulation ability and surface permeability. Moreover, Gd-containing host can also be used as promising X-ray tomography (CT) and magnetic resonance imaging contrast agent owing to the large X-ray absorption coefficient and paramagnetic properties.^42–44^ Therefore, it is significant importance for developing Gd-based materials with hollow-typed structure for multimodal bioimaging and drug delivery. However, most of the developed Gd-based hollow materials were used as only for drug delivery or optical bioimaging by using the traditional visible light, greatly impeding their application for *in vivo* deep-tissue and high sensitivity bioimaging due to the large absorption and scattering losses of visible right in biotissues. Gd-based hollow nanomaterials with combination of NIR-II bioimaging and drug delivery have not yet been explored. Therefore, it is significant importance to design a multifunctional hollow-typed nanoprobe by integrating both NIR-II optical bioimaging and drug delivery functions.

Herein, in order to integrate the dual-modal *in vivo* NIR-II/X-ray bioimaging and pH-responsive drug delivery into a system, we synthesized the yolk–shell-typed GdPO_4_:Nd^3+^ nanoprobes by a self-scarifying template method using cetyltrimethyl ammonium bromide (CTAB) as a structure-directing agent. Dual-modal *in vivo* NIR-II/X-ray bioimaging based on these GdPO_4_:Nd^3+^ spheres was performed. And the efficient pH-responsive drug delivery based on these probes was demonstrated. These results indicate that the yolk–shell-structured GdPO_4_:Nd^3+^ probes are promising agents for simultaneously combining multimodal bioimaging, especially for the new advanced NIR-II bioimaging with drug delivery to form synergistic theranostic platform.

## 2. Experimental

### 2.1 Chemicals and materials

All of the chemical reagents used in this experiment were obtained from commercial supplies and used as received without purification. GdCl_3_·6H_2_O and NdCl_3_·6H_2_O were all of 99.99% purity and purchased from QingDao elaborate Chemical Reagent Co. Ltd (Shandong). The corresponding rare-earth chlorides (RE)Cl_3_ solutions were obtained *via* dissolving RE chlorides into deionized (DI) water under stirring until the formation of a transparent solution with a designed concentration of 0.5 M. Doxorubicin (DOX, 99.5%) was purchased from Ekear Bio&Tech Co. Ltd (Shanghai). The other chemicals (analytical reagent) were ordered from Sinopharm Chemical Reagent Co., China.

### 2.2 Preparation of monodispersed Gd(OH)CO_3_:*x*% Nd^3+^ (*x* = 1.0, 2.0, 5.0) precursor

The monodispersed Gd(OH)CO_3_:*x*% Nd^3+^ (*x* = 1.0, 2.0, 5.0) precursor was prepared by urea-based homogeneous precipitation method by using urea as a precipitator according to the previous report.^34^ The as-prepared GdCl_3_ and NdCl_3_ solutions with a total amount of 1 mmol and a designed molar ratio of 99 : 1, 98 : 2, and 95 : 5 were dissolved in 50 mL DI water, respectively. Then, 2.0 g of urea (CO(NH_2_)_2_) was added into the former mixed solution and kept stirring for 2 h at room temperature. The resultant solution was then heated to 85 °C and reacted for 2 h in the oil bath with vigorously stirring. The resultant milky-white colloidal particles were collected *via* centrifugation, and washed with DI water and ethanol three times to obtain the monodispersed Gd(OH)CO_3_:*x*% Nd^3+^ (*x* = 1.0, 2.0, 5.0) precursor.

### 2.3 Synthesis of GdPO_4_:*x*% Nd^3+^ (*x* = 1.0, 2.0, 5.0) probe

The monodispersed GdPO_4_:*x*% Nd^3+^ (*x* = 1.0, 2.0, 5.0) spheres were prepared by a self-sacrificing template method.^34^ In a typical synthesis procedure, the as-prepared Gd(OH)CO_3_:*x*% Nd^3+^ (*x* = 1.0, 2.0, 5.0) precursor was dispersed into fresh DI water *via* ultrasonication for 30 min to acquire solution A. At the same time, 0.1 g of CTAB and 0.115 g of ammonium dihydrogen phosphate (NH_4_H_2_PO_4_) were added into 20 mL of fresh DI water with stirring to obtain a homogeneous solution B. After that, the solutions A and B were then mixed together and kept to stirring for 10 min. Subsequently, the as-obtained mixing solution was transferred into a stainless Teflon-lined autoclave, sealed and maintained at 200 °C for 24 h. Finally, after the autoclave was cooled to room temperature naturally, the final products settled at the bottom of the vessel were collected by centrifugation and removed the impurities *via* washing with DI water and ethanol three times.

### 2.4 Characterizations

The crystal phase structures of the Gd(OH)CO_3_:2% Nd^3+^ precursor and GdPO_4_:2% Nd^3+^ sphere samples were detected *via* powder X-ray diffraction (XRD) using a Rigaku D/max 2500 system at a scanning rate of 1° min^−1^ in the 2*θ* range of 10–80°, with Cu-Kα radiation (*λ* = 0.15406 nm) at 40 kV and 250 mA. The morphology, structure and size of these as-prepared samples were obtained by transmission electron microscopy (TEM, FEI Tecnai F20), and scanning transmission electron microscopy (STEM) equipped with the energy dispersive X-ray spectroscopy (EDS, Oxford Instrument) system using an accelerating voltage of 200 kV. The existing elements in the GdPO_4_:Nd^3+^ samples were further determined by X-ray photoelectron spectroscopy (XPS, Thermo Fisher Scientific Escalab 250Xi). The surface ligands of the samples were detected *via* a Fourier transform infrared spectrum (FTIR) by using a Magna 760 spectrometer (Nicolet). The NIR-II fluorescence spectra were recorded by using a NIRQuest512 spectrometer (Ocean Optics) under the excitation of 808 nm laser at room temperature. NIR-II fluorescence imaging of the as-prepared GdPO_4_:*x*% Nd^3+^ (*x* = 1.0, 2.0, 5.0) water solution was acquired by the InGaAs short-wavelength infrared (SWIR) detector (model: NIRvana™ camera system, default operating temperature: −80 °C, Princeton Instruments) with the excitation of 808 nm laser.

### 2.5 *In vivo* bioimaging in the NIR-II window

In order to assess the feasibility of GdPO_4_:2% Nd^3+^ probe for *in vivo* bioimaging in the NIR-II window, the 250 μL solution (2 mg mL^−1^) of sample was intravenously injected into an anesthetized Kunming mouse which intraperitoneally injected with 150 μL of 10 wt% pentobarbital sodium aqueous solution, and then *in vivo* NIR-II bioimaging at different time intervals from 1 h to 12 days after injection was acquired by the InGaAs SWIR detector (model: NIRvana™ camera system, default operating temperature: −80 °C, Princeton Instruments) under the excitation of 808 nm laser and the band-pass filter of 1200 nm to 1400 nm. All animal procedures in this study were performed in accordance with the Guidelines for Care and Use of Laboratory Animal Center of Hunan Normal University and approved by the Animal Ethics Committee of Hunan Province.

### 2.6 Evaluation of the contrast effect of *in vitro* and *in vivo* X-ray imaging

To evaluate the feasibility of GdPO_4_:2% Nd^3+^ probe for X-ray imaging, *in vitro* phantom X-ray imaging of the samples with different concentrations (0, 2.5, 5, 10, 20, 40 mg mL^−1^) was performed by a multi-modal *in vivo* imaging system (Bruker *In Vivo* FX Pro) under the operating voltage of 45 kV_^p^_, aluminum filter of 4 mm and exposure time of 30 s. The *in vitro* phantom X-ray imaging of iobitridol with different concentrations (0, 2.5, 5, 10, 20, 40 mg mL^−1^) was also acquired with the same method as described above for comparison.

To further investigate the feasibility of the GdPO_4_:2% Nd^3+^ probe as *in vivo* X-ray bioimaging contrast agents, another Kunming mouse was anesthetized, and then X-ray bioimaging of mouse injected with 250 μL of GdPO_4_:2% Nd^3+^ probe water solution (2 mg mL^−1^) was acquired by the same multi-modal *in vivo* imaging system under the aforementioned conditions.

### 2.7 *In vitro* loading and release of DOX

DOX loading into GdPO_4_:2% Nd^3+^ spheres was performed by mixing 2.5 mL of DOX phosphate buffer solution (PBS, 0.1 mg mL^−1^) with 7.5 mL of GdPO_4_:2% Nd^3+^ spheres (0.1 mg mL^−1^) with slow stirring at room temperature for 24 hours. After that, the resultant solution was centrifuged at 12 000 rpm for 8 min to separate the DOX-loaded spheres and the unbounded DOX. The DOX-loaded spheres were collected for further use and the clear liquid (containing ungrafted DOX) was retained for ultraviolet (UV)-visible absorbance spectrum assay to assess the content of DOX loaded in the GdPO_4_:Nd^3+^ spheres. The proportion of DOX loaded in the GdPO_4_:Nd^3+^ spheres was obtained by using the characteristic absorption peak at 480 nm of free DOX to subtract the absorption peak of the as-acquired clear liquid (contain of DOX) at the same wavelength.^45,46^ The UV-visible absorbance spectra were performed at a Lambda 750 UV/VIS spectrometer (PerkinElmer Inc.).

In order to study the release kinetics of DOX, the as-obtained DOX-loaded GdPO_4_:2% Nd^3+^ probe were dispersed in PBS (2 mL) at two pH values (5 and 7.4) for incubation at designed time intervals (0, 2, 4, 6, 8, 20, and 48 h). Then the PBS was centrifugally separated at 12 000 rpm for 4 min, and the supernatant liquid was maintained for UV-visible absorbance spectrum analysis. Then, the amounts of the released DOX in the supernatant liquid were calculated by the UV-visible absorbance spectrum.

### 2.8 Histological test

In order to assess the *in vivo* toxicity of GdPO_4_:2% Nd^3+^ sphere, histological test was performed. The GdPO_4_:2% Nd^3+^ spheres were injected into Kunming mice for 3 days and 7 days after intravenous injection to obtain the experimental groups. At the same time, the mouse without injection was denoted as the control group. Then, the heart, liver, spleen, lung, and kidney were collected from the experimental and control group, respectively. Subsequently, these organs were sliced and stained with hematoxylin and eosin (H&E) to monitor histological changes. And the histological sections were observed by an optical microscope.

## 3. Results and discussion

### 3.1 Structural characterization

**Scheme 1:**
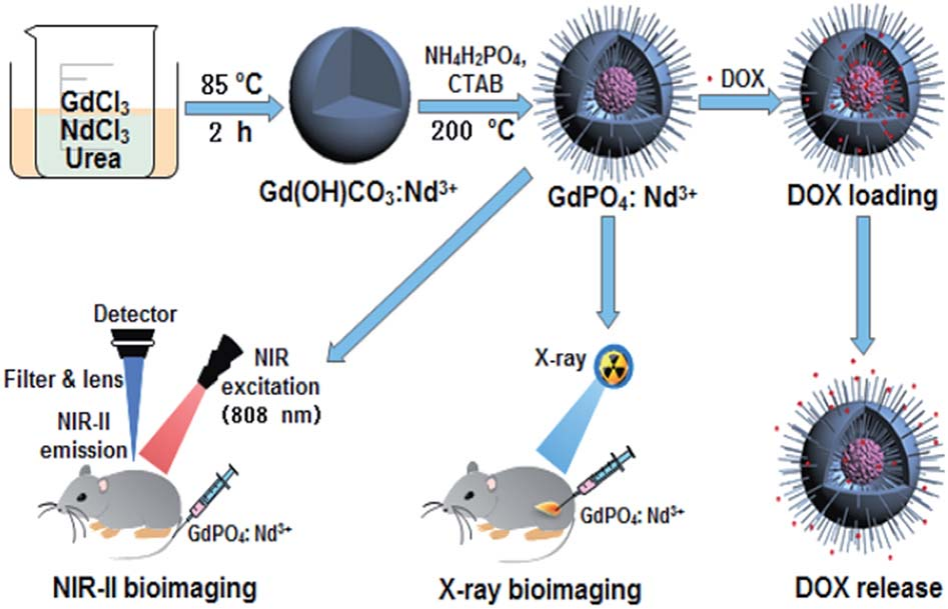
Schematic illustration of designing the theranostic GdPO_4_:Nd^3+^ nanoprobes for *in vivo* NIR-II/X-ray bioimaging, and pH-responsive drug delivery.

As shown in Scheme 1, the theranostic GdPO_4_:Nd^3+^ nanoprobes with dual-modal NIR-II/X-ray bioimaging and drug delivery functions were achieved by a self-scarifying template method from the precursor of Gd(OH)CO_3_:Nd^3+^.

**Fig. 1:**
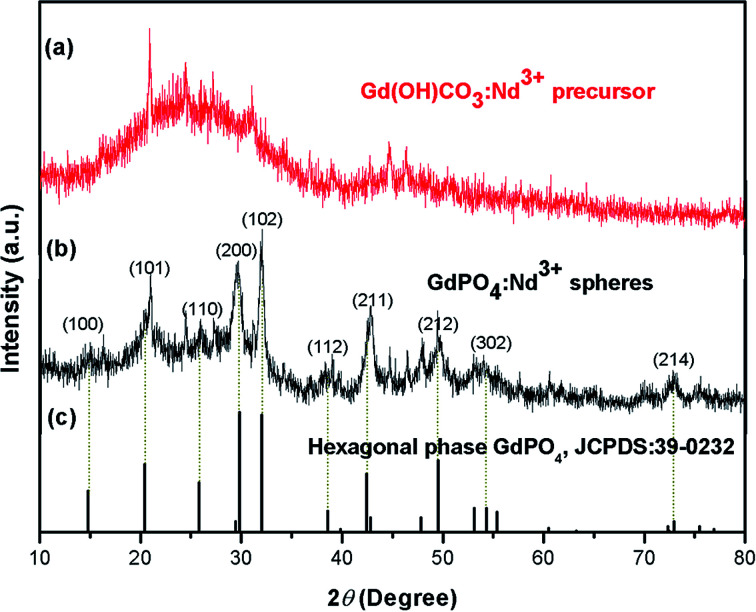
The XRD patterns of the as-prepared samples: (a) Gd(OH)CO_3_:2% Nd^3+^ precursor and (b) GdPO_4_:2% Nd^3+^ and (c) the standard hexagonal phase structure of GdPO_4_ (JCPDS no. 39-0232).

The crystal phases of the as-prepared samples were first analyzed by XRD (Fig. 1). As shown in the Fig. 1a, the XRD pattern of the precursor exhibits no obvious diffraction peak, indicating the amorphous structure of the precursor.^39^ While, the sharp and narrow diffraction peaks are observed for GdPO_4_:2% Nd^3+^ spheres, which can be well indexed to the standard hexagonal phase structure of GdPO_4_ (JCPDS no. 39-0232), indicating the formation of high crystallinity sample at relatively low hydrothermal temperature (200 °C).

**Fig. 2:**
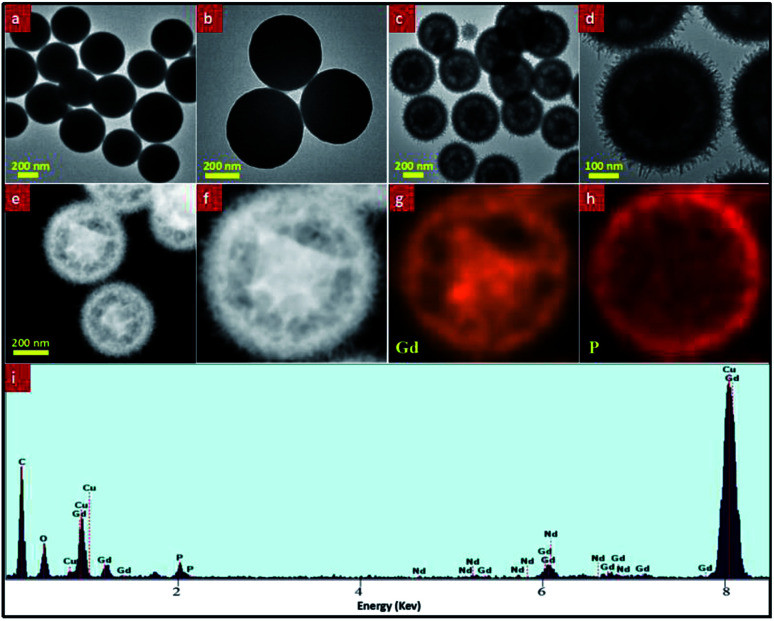
The typical TEM images of the as-prepared samples: (a) and (c) low-magnification TEM images of Gd(OH)CO_3_:2% Nd^3+^ and GdPO_4_:2% Nd^3+^, respectively; (b) and (d) high-magnification TEM images of Gd(OH)CO_3_:2% Nd^3+^ and GdPO_4_:2% Nd^3+^, respectively; (e) STEM image, (f–h) the corresponding STEM-EDS mapping of GdPO_4_:2% Nd^3+^, indicating the element distribution; (i) EDS result of GdPO_4_:2% Nd^3+^ sample.

The morphology and microstructure of the as-prepared precursor and final product were characterized *via* TEM (Fig. 2). TEM images show that both Gd(OH)CO_3_:2% Nd^3+^ precursor (Fig. 2a) and final product GdPO_4_:2% Nd^3+^ (Fig. 2c) are roughly spherical particles with high monodispersity. But as shown in the high-magnification TEM images of these two samples (Fig. 2b and d), it is obviously noted that the solid sphere of the Gd(OH)CO_3_:Nd^3+^ precursor (Fig. 2b) is changed to yolk–shell structure after hydrothermal treatment to form the final structure (Fig. 2d). To further confirm the formation of the yolk–shell structure, we have performed STEM, EDS mapping, and EDS line scan test. As shown in Fig. 2g and h and S1,† the Gd element is mainly distributed in the edge and middle of the sample, while the P element is mainly distributed on the edge of the sample, which clearly demonstrated that the un-reacted amorphous phase Gd(OH)CO_3_:Nd^3+^ precursor is located in the center of the GdPO_4_:Nd^3+^ sphere to form Gd(OH)CO_3_@GdPO_4_ yolk–shell structure. These TEM and STEM results reveal that the Gd(OH)CO_3_:Nd^3+^ precursor has not yet been fully changed to GdPO_4_ by hydrothermal treatment. It also should be pointed out that the un-reacted amorphous phase Gd(OH)CO_3_:Nd^3+^ precursor can be fully scarified by further prolonging hydrothermal time. Moreover, owing to the amorphous phase structure, the un-reacted Gd(OH)CO_3_:Nd^3+^ precursor located in the center of the sphere has no influence on the luminescent properties. In addition, compared with the Gd(OH)CO_3_ precursor, the surface of the final products GdPO_4_:2% Nd^3+^ becomes rough, which consists of numerous small nanorods (Fig. S2†). The EDS result (Fig. 2e) of the yolk–shell typed GdPO_4_:2% Nd^3+^ sample demonstrates the elemental composition of the final products, validating the existence of Gd, P, O, and Nd. And the XPS result of the as-prepared sample (Fig. S3†) also revealed the elemental composition of Gd, P, O, Nd, and C, further indicating the presence of unreacted Gd(OH)CO_3_:Nd^3+^ precursor.

In addition, the size of the nanoprobes can be readily controlled by adjusting the reaction parameters of the precursor Gd(OH)CO_3_. As shown in Fig. S4,† the size of GdPO_4_ spheres can be tuned by adjusting the size of the Gd(OH)CO_3_ precursor *via* modifying the reaction time and temperature. And the final size of GdPO_4_ probes can be tuned from 380–410 nm to 110–150 nm.

### 3.2 Analysis of FTIR

**Fig. 3:**
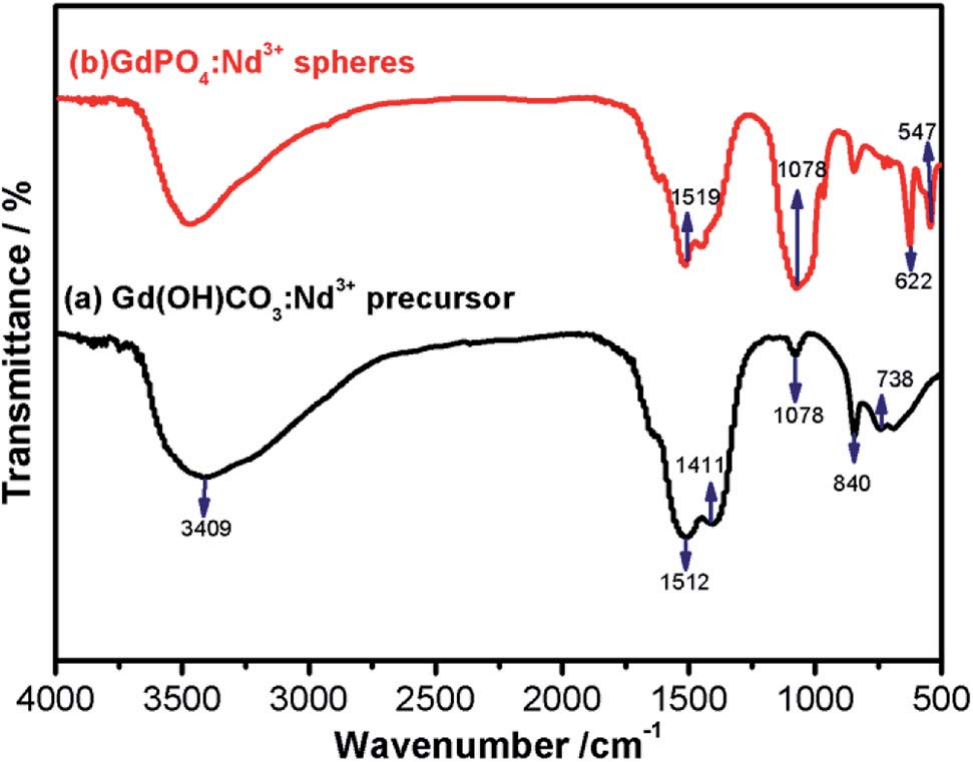
The FTIR spectra of the as-prepared samples: (a) Gd(OH)CO_3_:2% Nd^3+^ and (b) GdPO_4_:2% Nd^3+^.

In order to further examine the surface chemical ligands, the surface functional groups of the precursor and final products were identified *via* the FTIR spectra (Fig. 3). As shown in Fig. 3a, it is noted that the characteristic absorption bands of O–H (*ν*, 3409 cm^−1^; *δ*, 738 cm^−1^) and O–C–O (*ν*_as_, 1512 and 1411 cm^−1^; *ν*_s_, 1078 cm^−1^; *δ*, 840 cm^−1^), where the *ν*, *ν*_as_, *ν*_s_ and *δ* symbolised for stretching vibrations, asymmetric stretching vibrations, symmetric stretching vibrations and deformation, respectively.^47^ After hydrothermal reaction, the new vibration bands at 547 and 622 cm^−1^ and broadband peak centered at 1078 cm^−1^ were observed from the FTIR spectrum of GdPO_4_ (Fig. 3b, red line), matching well with the characteristic absorption of the phosphate groups and indicating the successful formation of GdPO_4_.^48^ It should be noted that the characteristic absorption bands around 1519 cm^−1^ in GdPO_4_:2% Nd^3+^ sample is also attributed to the asymmetric stretching vibrations of O–C–O, further implying that the presence of unreacted Gd(OH)CO_3_:2% Nd^3+^ precursor, which is consistent with XPS and EDS mapping results.

### 3.3 NIR-II luminescent properties

**Fig. 4:**
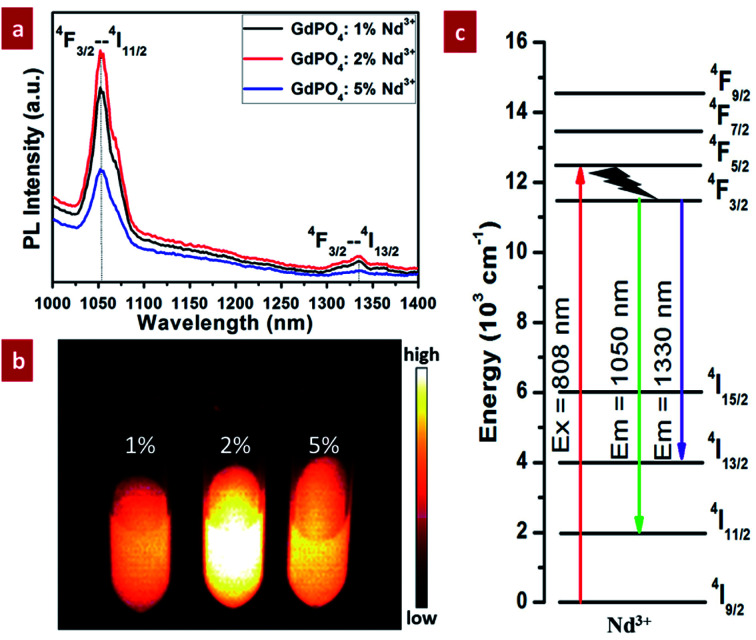
NIR-II photoluminescence properties of GdPO_4_:Nd^3+^ spheres under the excitation of 808 nm laser: (a) the emission spectra of the spheres doped with different contents of Nd^3+^; (b) the corresponding *in vitro* phantom NIR-II imaging of the GdPO_4_:Nd^3+^ solution; (c) schematic energy level diagram of Nd^3+^ for NIR-II emission.

NIR-II photoluminescence properties of GdPO_4_:Nd^3+^ probe were investigated under the excitation of 808 nm laser. As demonstrated in Fig. 4a, all of the samples doped with different Nd^3+^ present two characteristic NIR-II emission peaks: a dominant emission centered at 1050 nm and a weak emission centered at 1330 nm. And the emission peaks located at 1050 and 1330 nm are attributed to the ^4^F_3/2_–^4^I_11/2_ and ^4^F_3/2_–^4^I_13/2_ transition of Nd^3+^ (Fig. 4c), respectively. In addition, according to the emission spectra, the NIR-II luminescent intensity of GdPO_4_:Nd^3+^ can be significantly improved by increasing the content of Nd^3+^ from 1% to 2%. But it was weakened when the doping ratio of Nd^3+^ was increased to 5%, which was possibly due to the concentration quenching effect. The *in vitro* phantom NIR-II imaging of GdPO_4_ solutions doped with different Nd^3+^ (Fig. 4b) further reveal that the luminescent intensity is the strongest when doping 2% Nd^3+^, which is consistent with the results of the above spectral analysis. Based on this result, the subsequent *in vivo* NIR-II imaging was carried out by using the sample doped with 2% Nd^3+^.

On the other hand, the size of the nanoprobes has significant influence on the NIR-II luminescent properties, which subsequently affect the highly sensitive bioimaging application. Therefore, we also evaluated the size-dependent NIR-II emitting properties. As shown in Fig. S5,† the large sized (380–410 nm) GdPO_4_:2% Nd^3+^ probe presented the superior NIR-II emission than others. And, it should be noted that the GdPO_4_:2% Nd^3+^ probe with size of 110–150 nm made up of ultrasmall sized nanorods (Fig. S4†) presented the weakest NIR-II emitting intensity. Although the small sized nanoprobe is beneficial for bioapplications, the weakest NIR-II emission makes it unsuitable for high sensitivity NIR-II bioimaging. Thus, to achieve the optimal and highly sensitive NIR-II imaging, we choose the large sized GdPO_4_:2% Nd^3+^ probes (380–410 nm) for further bioimaging and drug delivery applications. It also should be pointed out that exploring small sized nanoprobe with improved NIR-II emission is significantly important, which needs further study.

### 3.4 *In vivo* imaging in the NIR-II window

**Fig. 5:**
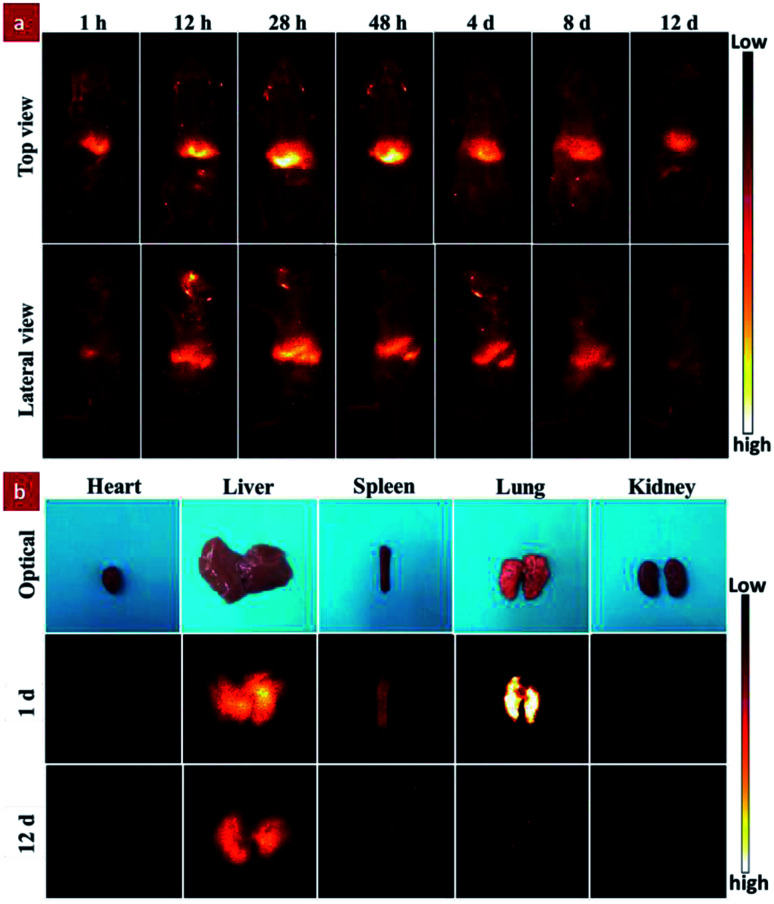
(a) *In vivo* NIR-II imaging-guided real-time tracking of GdPO_4_:2% Nd^3+^ probe in live mice at different injection times under the excitation of 808 nm laser; (b) the *ex vivo* imaging of the corresponding isolated organs of mice at different times after intravenous injection of the GdPO_4_:2% Nd^3+^ probe, including heart, liver, spleen, lung, and kidney.

To reveal the time-dependent biodistribution of the GdPO_4_:2% Nd^3+^ probe, NIR-II imaging-guided real-time tracking and *ex vivo* NIR-II fluorescence bioimaging were performed (Fig. 5). It should be noted that the mouse's food shows a remarkable NIR-II fluorescence at 1000–1100 nm spectral range under the excitation of the 808 nm laser.^12^ Moreover, nanoprobe capable of emitting 1.3–1.4 μm window is rationally used for *in vivo* NIR-IIa imaging owing to the significantly minimized scattering losses over wavelengths shorter than 1300 nm and decreased light absorption from water above 1400 nm.^49^ Therefore, all the *in vivo* and *ex vivo* NIR-II fluorescence bioimaging were detected in the 1200–1400 nm spectral range by using a band-pass filter (from 1200 nm to 1400 nm). As shown in Fig. 5a, in the top view, a remarkable NIR-II luminescent signal was preferentially observed in liver location after 1 h injection, and the signal was gradually enhanced with increasing the injection time. After 28 h injection, the signal was achieved at the maximum intensity and then gradually decreased. Furthermore, in the lateral view, a new signal was detected in the region of spleen and the signal was gradually enhanced after 12 h injection. Subsequently, the signal was gradually decreased after that and scarcely observed after 12 days of injection. According to the previous reports,^50–52^ the intravenously injected nanoprobes with negatively charged surface ligands were mainly accumulated at liver and spleen. As demonstrated by the aforementioned FTIR result, there are a lot of hydroxyl groups on the surface of the GdPO_4_:Nd^3+^ probe, so the surface of the GdPO_4_:Nd^3+^ probe presents the negatively charged nature in the water (Fig. S6†). From the *in vivo* bioimaging results, our developed GdPO_4_:Nd^3+^ probe also preferentially accumulate in the liver and spleen, which is consistent with the previous reports.^50–52^ Moreover, to further study the biodistribution of the GdPO_4_:2% Nd^3+^ probe in living mouse, the *ex vivo* imaging of the isolated organs collected from the dissected mice at different injection times was also performed. As demonstrated in Fig. 5b, the signals from the isolated organs were presented in the liver, lung and spleen, validating the same biodistribution trend and matching well with our previous report.^53^ Therefore, based on the above analysis, the GdPO_4_:2% Nd^3+^ probe can be used as a promising high sensitivity NIR-II contrast agent for *in vivo* dynamic fluorescence imaging.

### 3.5 *In vitro* and *in vivo* X-ray imaging

**Fig. 6:**
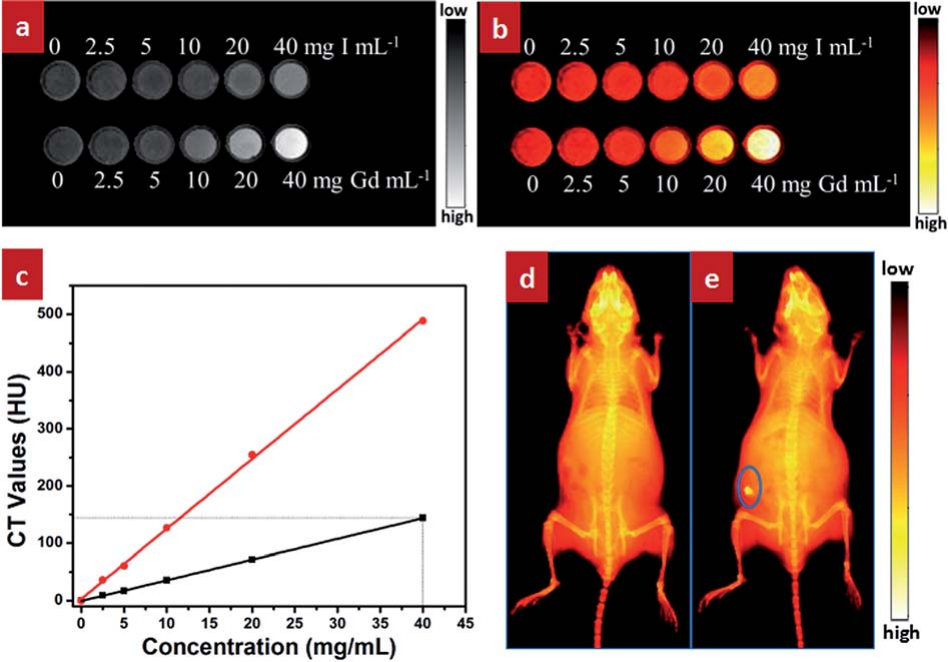
(a) *In vitro* phantom X-ray imaging of water solutions of GdPO_4_:2% Nd^3+^ spheres and iobitridol with different concentrations of Gd and I; (b) the corresponding pseudo-colored X-ray images of (a); (c) the measured Hounsfield units values of GdPO_4_:2% Nd^3+^ spheres (red circles) and iobitridol (black squares) as a function of the concentration of Gd (red lines) and I (black lines); *in vivo* X-ray bioimaging of Kunming mice: (d) without injection and (e) with subcutaneous injection of the GdPO_4_:2% Nd^3+^ solution.

Besides the excellent NIR-II optical bioimaging, these designed GdPO_4_:2% Nd^3+^ spheres can also be used as X-ray imaging agent owing to the large X-ray absorption coefficient of Gd^3+^.^42^ To evaluate the contrast effect of X-ray imaging, *in vitro* phantom X-ray imaging of different concentrations of GdPO_4_:Nd^3+^ spheres and iobitridol (a widely used X-ray imaging contrast agent) was performed. As shown in Fig. 6a and b, both X-ray absorption signals of GdPO_4_:Nd^3+^ spheres and iobitridol were gradually enhanced as increasing the concentration of the agent. Nevertheless, compared the CT Hounsfield units (HU) values (Fig. 6c) of the GdPO_4_:2% Nd^3+^ spheres with that of iobitridol, both agents exhibited a good linear correlation between the HU value and the concentration, but the HU values of the GdPO_4_:2% Nd^3+^ spheres were much higher than the iobitridol at equivalent concentrations, indicating the superior X-ray imaging ability of the GdPO_4_:Nd^3+^ spheres than iobitridol. To further assess *in vivo* X-ray bioimaging of the GdPO_4_:Nd^3+^ spheres, *in vivo* X-ray bioimaging of a Kunming mouse was acquired by using a multi-modal *in vivo* imaging system (Bruker *In Vivo* FX Pro) at a voltage of 45 kV_^p^_ after subcutaneously injected with the GdPO_4_:2% Nd^3+^ spheres. Compared with the untreated mouse (Fig. 6d), an obvious and high-contrast X-ray absorption signal located at the injected site (indicated by the blue circle) can be observed in the treated mouse. These results reveal that the as-prepared GdPO_4_:2% Nd^3+^ spheres can be used as promising agent for X-ray imaging.

### 3.6 Drug loading and release

**Fig. 7:**
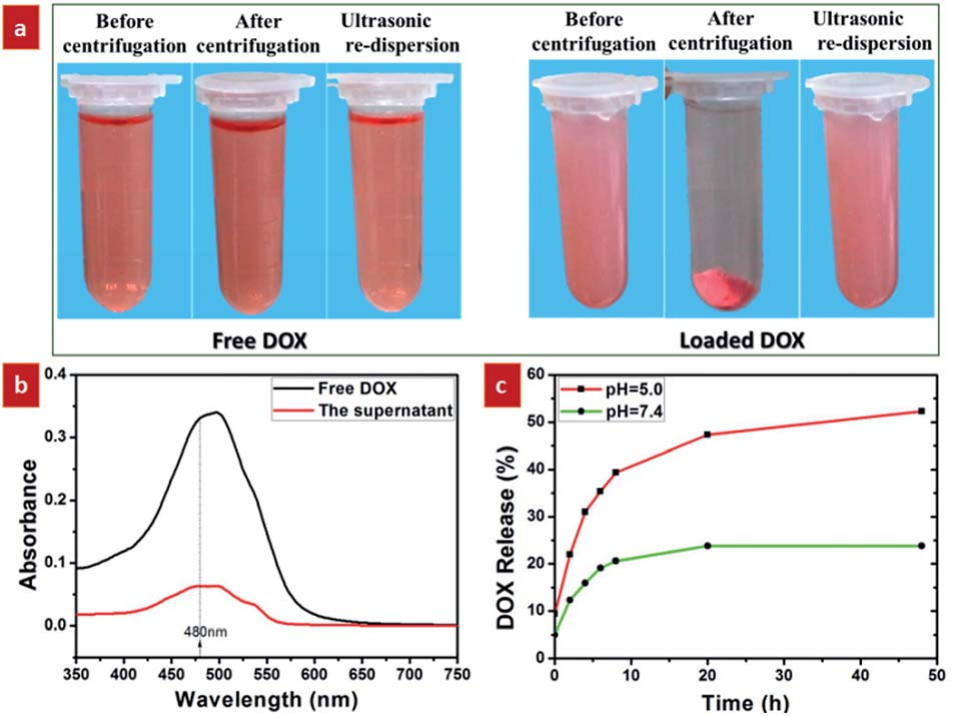
pH-responsive DOX-loading and releasing properties: (a) digital photographs of free DOX and DOX-loaded samples after centrifugation and ultrasonication; (b) UV-vis spectra of free DOX (black) and the supernatant transparent liquid of centrifuged DOX-loaded solution (red); (c) pH-responsive DOX release from the DOX-loaded samples over time in PBS at pH values of 5.0 (red) and 7.4 (green).

As demonstrated by the aforementioned FTIR result and Fig. S6,† the surface of the GdPO_4_:Nd^3+^ sphere presents the negatively charged in the pH = 7.4 due to the presence of a lot of hydroxyl groups on the surface. Therefore, these negatively charged GdPO_4_:Nd^3+^ spheres can easily interact with the positively charged DOX molecules through electrostatic interaction.^36^ On the other hand, the large internal space of the as-prepared yolk–shell spheres can be used as ideal structure for drug delivery. Therefore, in order to evaluate the drug delivery application of the as-prepared yolk–shell spheres, the DOX was chosen as a model drug for the loading and pH-responsive release behaviours. Firstly, the DOX-loaded solution was obtained by continuously stirring the mixed solution containing the GdPO_4_:2% Nd^3+^ spheres and DOX at room temperature for 24 h. To verify the ability of loading of DOX in the GdPO_4_:Nd^3+^ spheres, the comparison experiment for the DOX-loaded and free DOX solution was performed by centrifugation and ultrasonication (Fig. 7a). As shown in Fig. 7a, the DOX-loaded solution exhibits a weaker red color than the free DOX solution. And after centrifugation, the DOX-loaded solution was turned to form the reddish precipitate and nearly transparent supernatant liquid. While, the free DOX solution presented no significant color change and no obvious precipitate was observed. These findings unambiguously indicate that the DOX is successfully loaded into the GdPO_4_:Nd^3+^ spheres. Furthermore, the characteristic absorption peak at 480 nm of the free DOX and supernatant transparent liquid of the centrifuged DOX-loaded solution was detected by UV/vis spectrometer. As shown in Fig. 7b, the characteristic absorption peak intensity of the free DOX was higher than the absorption peak of the supernatant transparent liquid, further validating the successfully loading DOX into the GdPO_4_:Nd^3+^ sphere.

Moreover, the tumor tissues also present a more acidic microenvironment in comparison to the physiologically neutral pH of normal tissues and bloods.^5^ Thus, developing pH-responsive drug delivery system was beneficial to improving the therapeutic efficacy of the tumor and reducing the side effects of the drugs. Here, we selected pH values of 5 and 7.4 to study the pH-dependent drug release efficiency. As shown in Fig. 7c, at pH = 7.4, only 23.8% of the DOX was released from the GdPO_4_:Nd^3+^ sphere after 20 h. By contrast, it only takes less than 4 h to reach a comparable level of DOX release at pH = 5. This demonstrated that DOX was released slowly in the neutral environment and released rapidly in acidic environment, which was mainly ascribed to the weakened electrostatic interactions under acidic conditions by the promoted protonation of hydroxyl groups on the surface.^5,36^ To further validate these pH-responsive mechanism, the zeta potential (Fig. S6†) of the GdPO_4_:2% Nd^3+^ samples at pH = 5.0 was detected. As demonstrated, the surface charge was changed from negative to positive by adjusting the pH value from 7.4 to 5.0, further validating the protonation of hydroxyl groups on the surface to form positively charged surface under acidic microenvironment, subsequently weakening electrostatic interactions and promoting the pH-responsive drug release, which is consistent with previous reports.^5,36^ This result demonstrates that the developed yolk–shell structured GdPO_4_:2% Nd^3+^ probes are promising pH-responsive drug delivery system.

### 3.7 *In vivo* toxicity analysis

**Fig. 8:**
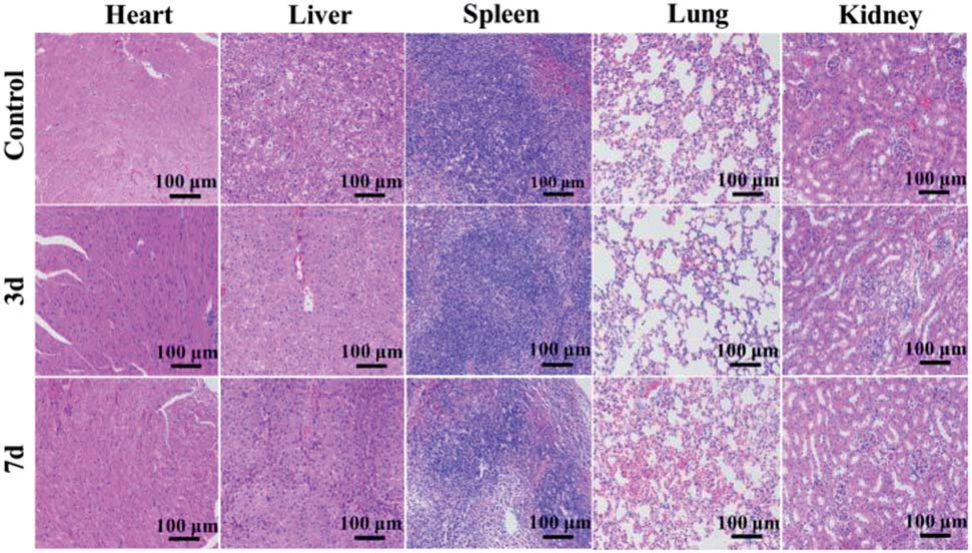
*In vivo* toxicity tests of H&E-stained tissue sections obtained from mice without injection, 3 and 7 days after injection of the GdPO_4_:2% Nd^3+^ sample.

*In vivo* toxicity of GdPO_4_:2% Nd^3+^ samples was evaluated *via* histological test of the histopathological changes in heart, liver, spleen, lung, and kidney. As shown in Fig. 8, after 3 and 7 days injection, no noticeable tissue damage and inflammatory lesions are detected in comparison with the control group, implying the low toxicity and good biocompatibility of GdPO_4_:2% Nd^3+^ spheres *in vivo*.

## 4. Conclusions

In conclusion, the multifunctional luminescent agents, hexagonal phase Nd^3+^ doped GdPO_4_ spheres with yolk–shell structure, were developed for dual-modal *in vivo* NIR-II/X-ray bioimaging and pH-responsive drug delivery. These Nd^3+^ doped GdPO_4_ spheres present excellent NIR-IIa emission centered at 1330 nm under the excitation of 808 nm laser. Moreover, the biodistribution and real-time tracking of GdPO_4_:Nd^3+^ spheres in living mice were successfully demonstrated by *in vivo* NIR-IIa bioimaging, showing that these probes were mainly accumulated in liver and spleen. Owing to the large X-ray absorption coefficient of Gd^3+^, the *in vitro* phantom and *in vivo* X-ray imaging revealed that the developed GdPO_4_:Nd^3+^ probes were also superior X-ray imaging agent than the commonly used iobitridol. More importantly, apart from the excellent dual-modal bioimaging, the yolk–shell structured GdPO_4_:Nd^3+^ probe was also used as an excellent pH-responsive drug carrier. The DOX release ratio was calculated to be 40% within 10 h at pH = 5, demonstrating the potential application in targeted therapy of tumor. Therefore, our findings open up the possibility of designing the new advanced theranostic nanoplatform by integrating the next generation NIR-IIa optical bioimaging and drug delivery function.

## Conflicts of interest

There are no conflicts to declare.

## Supplementary Material

RA-008-C7RA12864A-s001
