# Supplementary material for: Short-wave near-infrared emissive GdPO4:Nd3+ theranostic probe for in vivo bioimaging beyond 1300 nm
Source: RSC Adv. 2018 Apr 4;8(23):12832–40. doi: 10.1039/c7ra12864a (PMC9079334; doi:10.1039/c7ra12864a)
Supplement: RA-008-C7RA12864A-s001 [file RA-008-C7RA12864A-s001.pdf]

Electronic Supplementary Material (ESI) for RSC Advances.
This journal is © The Royal Society of Chemistry 2018

Supporting Information

Short-wave near-infrared emissive $GdPO_4$:Nd^{3+} theranostic probe for *in vivo* bioimaging beyond 1300 nm

Qiuhua Yang,[a] *Xiaolong Li,*[a] *Zhenluan Xue,*[a] *Mingyang Jiang,*[a] *Youbin Li,*[a] *SongjunZeng,*[*a]

[a] College of Physics and Information Science, Key Laboratory of Low-dimensional Quantum Structures and Quantum Control of the Ministry of Education, Synergetic Innovation Center for Quantum Effects and Applications, Hunan Normal University, Changsha, 410081, Hunan, China.

Email: songjunz@hunnu.edu.cn

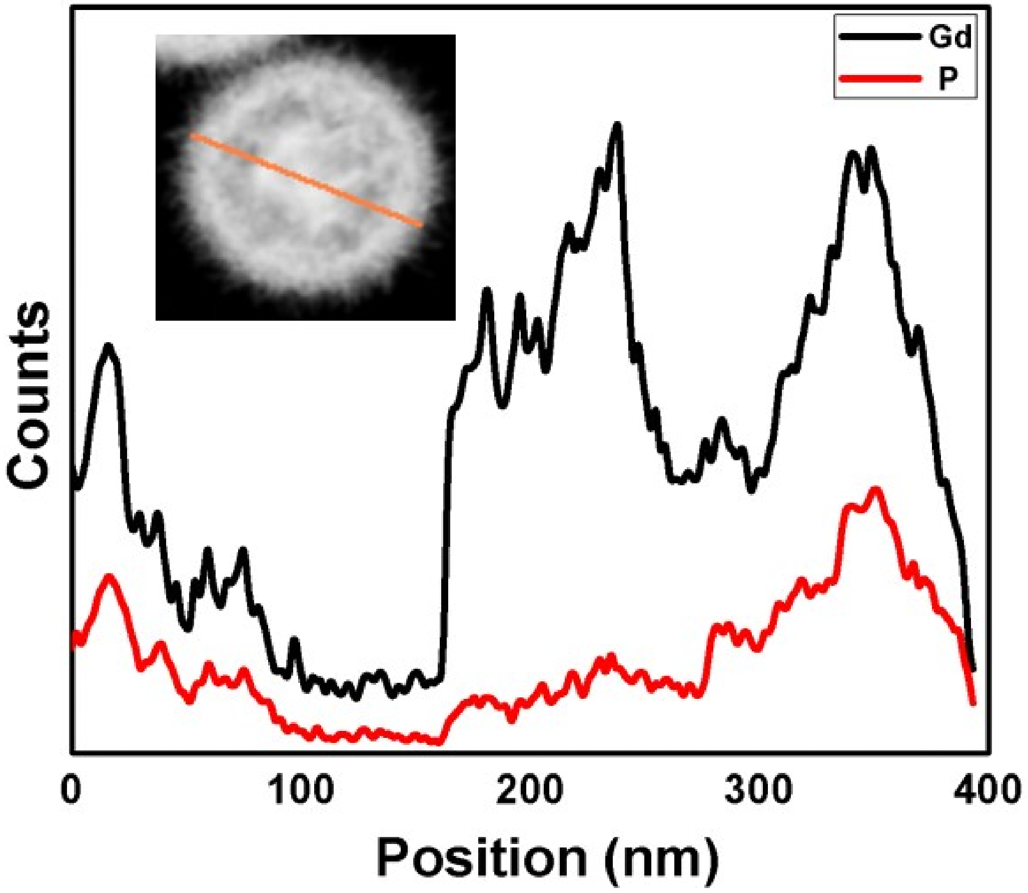

Fig S1 The EDS line scan along the line in the STEM image (inset) of $GdPO_4$:Nd^{3+}.

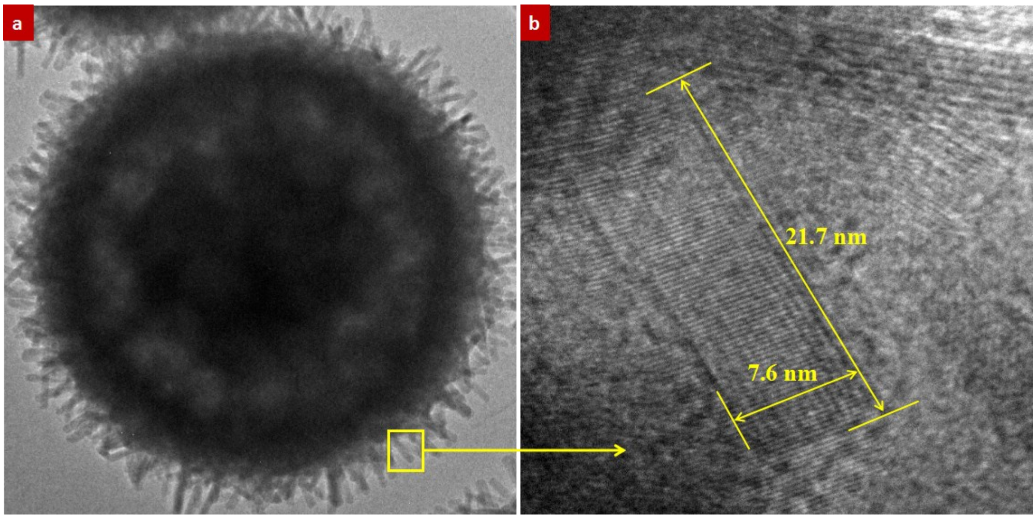

Fig. S2 TEM images of $GdPO_4$:Nd^{3+}: (a) the high-magnification TEM image; (b) the high-resolution TEM image taken from yellow rectangle of (a).

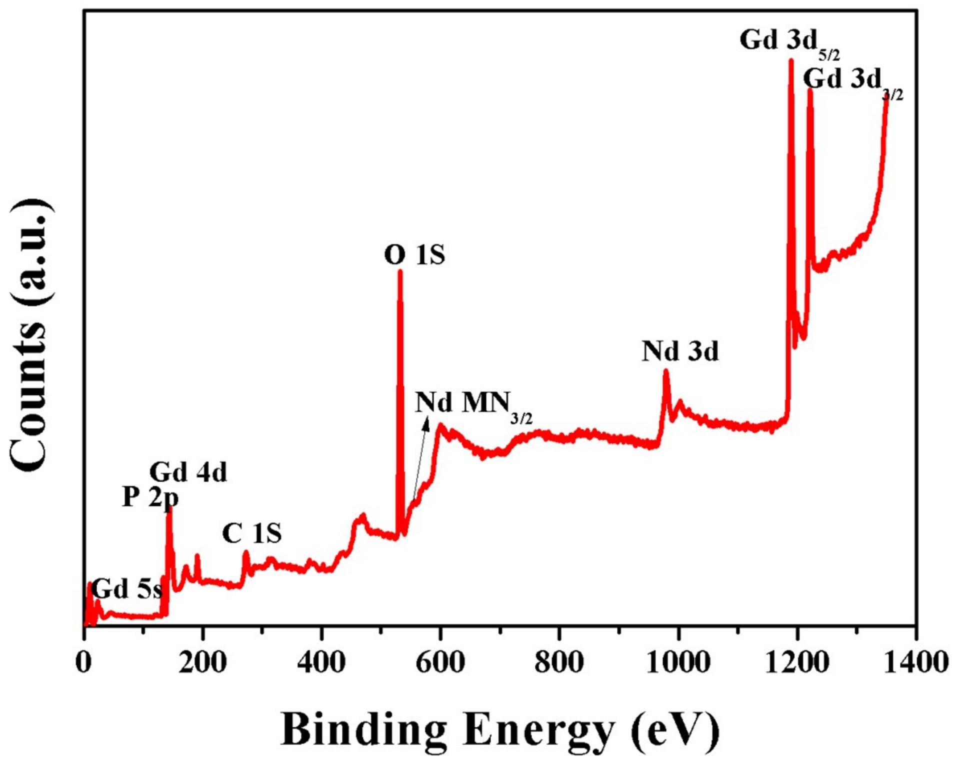

Fig. S3 The XPS spectra of the as-synthesizedGdPO$_4$: 2% Nd^{3+}.The 5s level of Gd at 38 eV, 2p level of P at 142 eV, 4d level of Gd at 143 eV, 1S level of C at 286.6 eV, 1S level of O at 532 eV, MN$_{3/2}$ level of Nd at 556 eV, 3d$_{5/2}$ and 3d$_{3/2}$ levels of Nd at 979 and 1003 eV, 3d$_{5/2}$ and 3d$_{3/2}$ level of Gd at 1189 and 1221 eV.

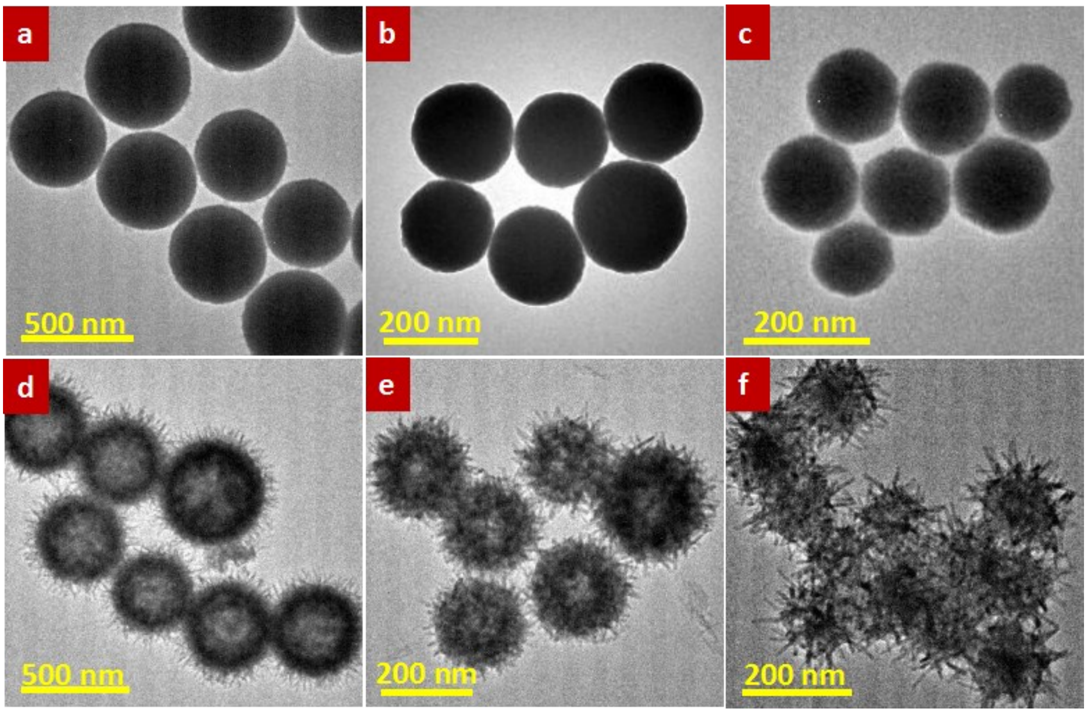

Fig. S4 The typical TEM images of the as-prepared samples: (a) and (d) are $Gd(OH)CO_3$: 2% Nd^{3+} precursor and $GdPO_4$: 2% Nd^{3+} samples with size of 380-410 nm, respectively; (b) and (e) are $Gd(OH)CO_3$: 2% Nd^{3+} precursor and $GdPO_4$: 2% Nd^{3+} samples with size of 200-220 nm, respectively; (c) and (f) are $Gd(OH)CO_3$: 2% Nd^{3+} precursor and $GdPO_4$: 2% Nd^{3+} samples with size of 110-150 nm, respectively.

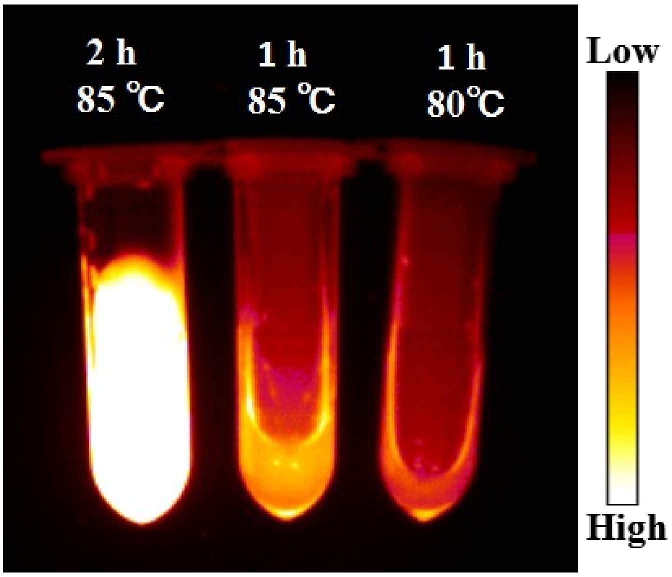

Fig. S5 *In vitro* phantom NIR-II imaging of the $GdPO_4$: 2% Nd^{3+} solution with different size under the excitation of 808 nm laser. left: 380-410 nm, middle: 200-220 nm, right: 110-150 nm.

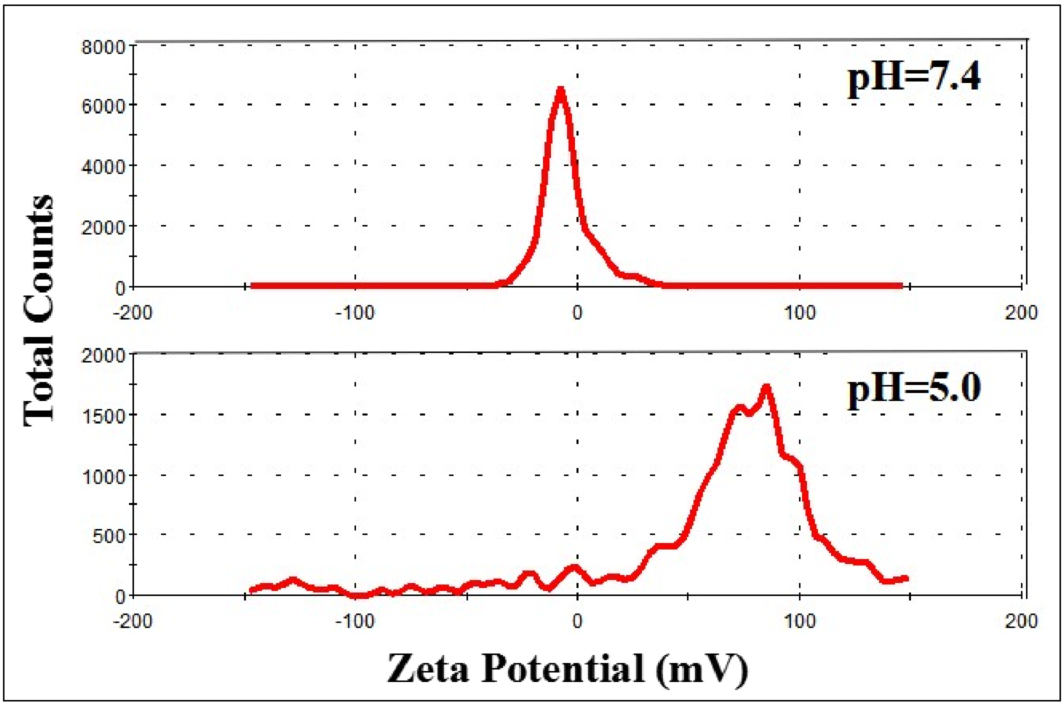

Fig. S6 Zeta potential of $GdPO_4{:}Nd^{3+}$ under different pH values.
